# A ceRNA approach may unveil unexpected contributors to deletion syndromes, the model of 5q- syndrome

**DOI:** 10.18632/oncoscience.261

**Published:** 2015-11-11

**Authors:** Walter Arancio, Swonild Ilenia Genovese, Lucia Bongiovanni, Claudio Tripodo

**Affiliations:** ^1^Biomedical Department of Internal and Specialized Medicine, Palermo University School of Medicine, Palermo, Italy; ^2^Tumour Immunology Unit, Human Pathology Section, Department of Health Science, Palermo University School of Medicine, Palermo, Italy

**Keywords:** CeRNA, competing endogenous RNAs, genomic deletions, 5q- syndrome, myelodysplastic syndrome

## Abstract

In genomic deletions, gene haploinsufficiency might directly configure a specific disease phenotype. Nevertheless, in some cases no functional association can be identified between haploinsufficient genes and the deletion-associated phenotype. Transcripts can act as microRNA sponges. The reduction of transcripts from the hemizygous region may increase the availability of specific microRNAs, which in turn may exert *in-trans* regulation of target genes outside the deleted region, eventually contributing to the phenotype. Here we prospect a competing endogenous RNA (ceRNA) approach for the identification of candidate genes target of epigenetic regulation in deletion syndromes.

As a model, we analyzed the 5q- myelodysplastic syndrome. Genes in haploinsufficiency within the common 5q deleted region in CD34+ blasts were identified *in silico*. Using the miRWalk 2.0 platform, we predicted microRNAs whose availability, and thus activity, could be enhanced by the deletion, and performed a genomewide analysis of the genes outside the 5q deleted region that could be targeted by the predicted miRNAs. The analysis pointed to two genes with altered expression in 5q- transcriptome, which have never been related with 5q- before.

The prospected approach allows investigating the global transcriptional effect of genomic deletions, possibly prompting discovery of unsuspected contributors in the deletion-associated phenotype. Moreover, it may help in functionally characterizing previously reported unexpected interactions.

## INTRODUCTION

The vast majority of human malignancies exhibits chromosomal rearrangements. These rearrangements span from deletions, duplication, balanced and unbalanced translocations, to the gain or loss of whole chromosomes, which are often associated [1–8].

Frequently, specific genomic rearrangements are associated with specific malignant phenotypes.

One notable example is the Philadelphia chromosome being the hallmark of chronic myelogenous leukemia (CML). Philadelphia chromosome is the result of a balanced translocation involving chromosomes 9 and 22 [9]. The final product of this rearrangement is the production of a chimerical protein (p210^BCR-ABL^) with constitutive tyrosine kinase activity, which is responsible for the CML clone expansion [10].

The study of the effects of translocations usually leads to the identification of genes at the breakpoints that gain or lose functions and that are causative of the phenotypes observed.

Differently, the study of genomic deletions or duplications is less straightforward because of the rarity of homozygous deletions and since the involved regions are commonly gene-rich.

The main efforts to elucidate the effects of deletions have been focused on the study of every single gene coded within the deleted region. The rationale is that if a gene shows haploinsufficiency, a reduced amount and activities of the gene products can contribute to the phenotype [11–35].

For example, in Williams syndrome the 7q11.23 band is deleted. The deleted region includes more than 25 genes, comprising the *ELN* gene. *ELN* gene codes for the elastin protein, and its haploinsufficiency is associated with the typical cardiovascular abnormalities of the syndrome [36].

Interestingly, very often this axiomatic relationship between genic deletion and phenotype is not easily identified. This is the case of 5q- syndrome [9], which we have adopted as a model to test a novel *in silico* approach to investigate the global effect of deletions. The 5q-syndrome is a hematological disorder characterized by the loss of the 5q31.1 band in bone marrow hematopoietic cells. This chromosome abnormality usually leads to a myelodysplastic syndrome (MDS) that can also evolve towards acute myeloid leukemia (AML) [37-42]. In the commonly deleted region of 5q-, several genes have been suggested to play a role in the syndrome, such as *SPARC*, *RPS14* and *hsa-miR-145*, all of them contributing to specific features of the 5q- myelodysplasia. However, the reduced activity of such genes does not explain every facet of the complex phenotype of 5q- syndrome [37–42].

In order to investigate the global effect of the 5q deletion, we prospected a competing endogenous RNAs (ceRNA) approach. CeRNA rationale relies on the consideration that RNA transcripts regulate one another by competing for shared microRNAs [43-48]. The loss or haploinsufficiency of a specific gene can free a certain amount of regulating microRNAs that, in turn, can act *in-trans* to regulate a subset of other transcripts. CeRNA approach has given interesting results both in oncological and non-oncological diseases. Usually, competing RNAs are explored using a single bait gene, as in the case of *PTEN* [49, 50], *LMNA* [51, 52], *SOX*2 [53], *hTERT* [54]. To the best of our knowledge, the effect of the loss of a pool of genes, as in the case of a deletion, using a ceRNA approach, has never been investigated before.

In 5q- syndrome, we selected, by *in silico* analyses, a set of microRNAs that might be freed by the haploinsufficiency of the genes coded within the deleted region, and identified the genes that could be regulated by the microRNA set as a whole.

This approach, which extends the research for ceRNAs from a single bait gene, to a set of genes, allows identifying those genes whose activity can be perturbed by a genomic deletion, considered as a whole. Notably, it could provide an explanation to the phenotypes observed in syndromes caused by deletions, independently from the genes coded within the deleted region.

## RESULTS AND DISCUSSION

Over the last few years, it has become clear that different RNA species can cross-talk and regulate one another [43–48, 55–59]. Due to a hemizygosis condition, several species of RNAs can be downregulated compared to a wild type condition. This global loss of transcripts might have an impact on the RNA-mediated cellular systems of regulation. In particular, we investigated if this loss of transcripts could have an impact on microRNA-mediated systems of regulation. MicroRNAs are small non-coding RNAs that regulate the gene expression, mostly at a post-transcriptional level [60–64]. In particular, RNA transcripts can regulate one another by competing for shared microRNAs. The RNAs that regulate one another in this way are called competing endogenous RNAs or ceRNAs. Competitive endogenous RNAs cross-regulation involves sequestration of shared microRNAs and gives rise to rather complex regulatory networks [43–48].

The loss of several transcripts at once during a deletion might free a sufficient amount of microRNAs that can assert a detectable effect *in trans* outside from the deleted region. Moreover, if several genes in haploinsufficiency within the deleted region are regulated by the same set of microRNAs, we might be able to identify a deletion-specific signature characterized by an increased activity of specific microRNAs.

In brief, a deletion could have an impact on the activity of a specific set of microRNAs that may in turn alter the activity of genes outside the deleted region and apparently unrelated with the genomic deletion. This alteration might contribute to determine the phenotypes of deletion syndromes.

**Figure 1:**
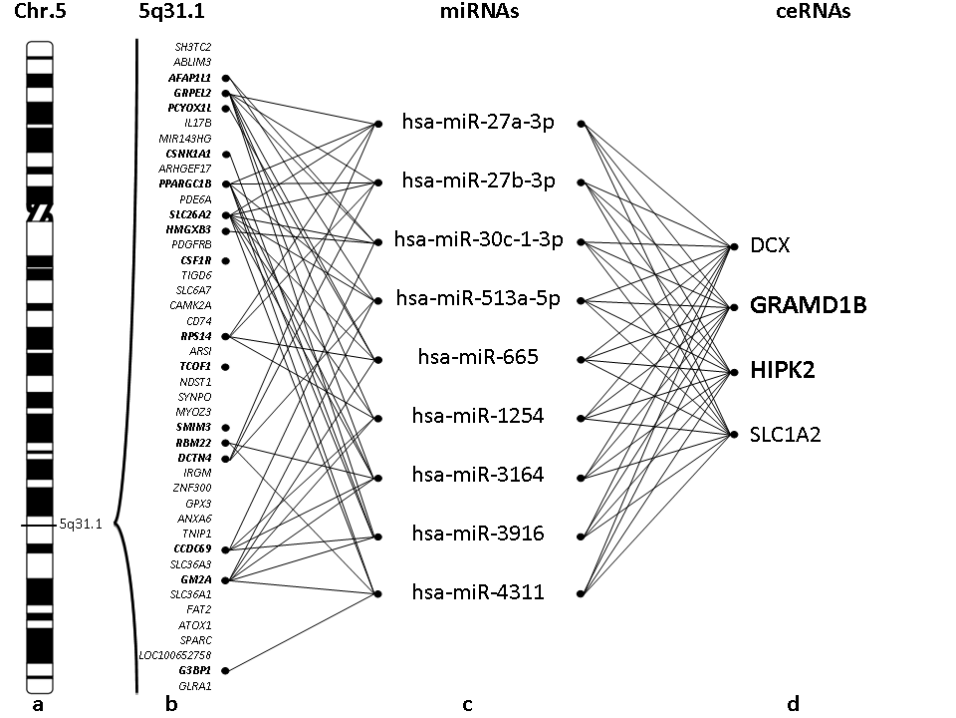
Schematic representation of the ceRNA analysis on 5q deletion **a.** The ideogram of human 5 chromosome (modified from David Adler); **b.** The genes within the common deleted 5q- region (the genes in haploinsufficiency are in bold); **c.** The microRNAs that regulated 5 or more genes in haploinsufficiency in b; **d.** The genes that are regulated by the 9 microRNAs in c and that are considered the putative ceRNAs (in bold the genes that show a significant different expression in 5q- patients compared with the controls).

In order to test our hypothesis, we used the 5q-syndrome model to investigate if a ceRNA approach could be useful to identify unexpected contributors to deletion syndromes. The approach adopted is graphically summarized in Figure 1.

We took advantage of the published GDS3795 affymetrix array dataset [65], which collects the global gene expression profiling of bone marrow CD34+ cells of myelodysplastic syndrome patients and healthy controls.

We first identified the patients with 5q deletion as the only reported genomic abnormality (see Supplemental data), and then, using their expression data, we selected the genes in haploinsufficiency within the common 5q deleted region, which are listed in Table 1.

**Table 1:** Identification of haploinsufficient genes within the commonly deleted region of 5q-

Gene name	Mean 5q-	SD 5q-	SE 5q-	Mean CNTR	SD CNTR	SE CNTR	p-value	p <0.05
ABLIM3	48.59779	58.31375	10.82859	22.49942	4.97781	1.2073	**0.07359**	
AFAP1L1	12.05416	3.3277	0.61794	15.09705	3.75227	0.91006	**0.00653**	**DOWN**
ANXA6	122.24087	67.11685	12.46329	147.35532	52.42362	12.7146	**0.19289**	
ARHGEF17	147.6068	37.55099	6.97304	133.18229	28.94435	7.02004	**0.18011**	
ARSI	19.25328	3.34174	0.62055	20.23331	7.4089	1.79692	**0.54062**	
ATOX1	193.08972	81.26299	15.09016	211.23735	30.26857	7.34121	**0.38248**	
CAMK2A	32.69334	10.36123	1.92403	32.51296	10.37561	2.51645	**0.95483**	
CCDC69	63.50043	31.51593	5.85236	107.38927	32.08788	7.78245	**0.00004**	**DOWN**
CD74	38.76174	8.96062	1.66395	40.6401	7.54131	1.82904	**0.47179**	
CSF1R	79.69017	44.38093	8.24133	106.02755	38.34201	9.2993	**0.04748**	**DOWN**
CSNK1A1	59.94675	54.80374	10.1768	101.51224	49.24745	11.94426	**0.01347**	**DOWN**
DCTN4	47.74753	16.37672	3.04108	68.13771	18.98236	4.6039	**0.00039**	**DOWN**
FAT2	19.86582	3.65128	0.67803	20.04196	3.26452	0.79176	**0.87047**	
G3BP1	57.54757	22.68119	4.21179	83.12785	23.39945	5.6752	**0.00069**	**DOWN**
GLRA1	12.83507	1.82292	0.33851	12.48884	1.03018	0.24986	**0.4773**	
GM2A	36.88007	10.41824	1.96886	46.9379	9.92848	2.40801	**0.00262**	**DOWN**
GPX3	36.37417	33.64503	6.24773	37.93556	27.76831	6.73481	**0.87237**	
GRPEL2	64.30569	23.59284	4.38108	98.69718	30.05175	7.28862	**0.00009**	**DOWN**
HMGXB3	92.76827	22.19641	4.12177	129.66734	24.83669	6.02378	**0.000005**	**DOWN**
IL17B	23.57699	3.80584	0.70673	25.38715	2.49688	0.60558	**0.08732**	
IRGM	8.72469	1.41946	0.26359	9.03116	0.93882	0.2277	**0.43232**	
LOC100652758	Data not available							
MIR143HG	45.98064	7.97738	1.48136	44.04348	5.67301	1.37591	**0.38484**	
MYOZ3	126.92361	34.89592	6.48001	138.42359	30.43793	7.38228	**0.26498**	
NDST1	60.03087	27.17387	5.04606	58.14676	23.76045	5.76275	**0.81347**	
PCYOX1L	117.69138	49.35714	9.16539	166.65525	69.23529	16.79202	**0.0077**	**DOWN**
PDE6A	23.06221	12.30975	2.28586	20.27092	2.3138	0.56118	**0.36191**	
PDGFRB	23.11002	7.09533	1.31757	23.33169	3.82961	0.92882	**0.90605**	
PPARGC1B	44.98414	17.74701	3.29554	85.30953	26.02103	6.31103	**0.0000001**	**DOWN**
RBM22	63.53639	26.10673	4.8479	94.33986	17.52159	4.24961	**0.00009**	**DOWN**
RPS14	7856.9131	1234.65184	229.26909	10598.78235	941.02851	228.23294	**0.0000000006**	**DOWN**
SH3TC2	14.87956	4.13834	0.76847	14.6208	3.05314	0.7405	**0.82371**	
SLC26A2	114135	53.33529	9.90411	200.29804	70.23587	17.0347	**0.00003**	**DOWN**
SLC36A1	44.44143	14114	2.6209	46.69696	15.60091	3.78378	**0.61728**	
SLC36A3	Data not available							
SLC6A7	46.04152	6.0099	1.11601	47.21034	6.08871	1.47673	**0.52959**	
SMIM3	260.38469	141.71754	26.31629	500.79865	310.45128	75.2955	**0.00081**	**DOWN**
SPARC	49.30088	41.99314	7.79793	62.24083	28.91624	7.01322	**0.26807**	
SYNPO	110.19731	20.31167	3.77178	115.67666	15.80301	3.83279	**0.34516**	
TCOF1	57.14866	25.18242	4.67626	73.23271	19.88939	4.82388	**0.02946**	**DOWN**
TIGD6	7.45674	0.79702	148	7.39382	0.78457	0.19029	**0.79614**	
TNIP1	139.14645	86.98013	16.1518	154.56547	45.26099	10.9774	**0.50195**	
ZNF300	96.84063	73.90228	13.72331	86.83198	35.88454	8.70328	**0.60446**	

A comparison of the normalized expression levels, as reported in GDS3795 dataset, of the genes within the common deleted region between 5q- patients and controls. The genes that showed in a student’s t-test a statistically significant (p<0.05) reduction of expression levels in 5q- specimens were considered as haploinsufficient genes. SE= Standard Error. SD= Standard Deviation. CNTR= Controls.

Using the bioinformatics approach described in the Methods section, we identified a set of microRNAs that putatively regulate the genes in haploinsufficiency. Each gene was regulated by a different set of microRNAs, but overall some microRNAs regulate a larger set of genes. Organizing those microRNAs in a hierarchical order, we were able to identify the most represented microRNAs. MicroRNAs that putatively regulated at least 5 of the haploinsufficient genes within the 5q- deleted region were selected. These included: hsa-miR-3164, hsa-miR-513a- 5p, hsa-miR-30c-1-3p, hsa-miR-1254, hsa-miR-3916, hsa-miR-27a-3p, hsa-miR-27b-3p, hsa-miR-4311, and hsa-miR-665 (see Supplemental data).

We then looked for genes that were predicted to be regulated by all of the 9 microRNAs, the rationale being that if these microRNAs could not bind a fraction of their natural targets due to the haploinsufficiency of the 5q-coded genes, they were free to exert their control on the remaining targets, deregulating the control network.

The analysis pointed out 4 genes, namely *DCX*, *GRAMD1B*, *HIPK2* and *SLC1A2*, which were putatively regulated by all the 9 microRNAs. Among these genes, *GRAMD1B and HIPK2,* showed significantly different mRNA expression in 5q- CD34+ cells as compared with control CD34+ cells in the same GDS3795 dataset, being significantly down- and up-regulated, respectively. Of note, the two genes that did not show significant variation between 5q- and control CD34+ cells both showed very low expression levels (Table 2).

**Table 2:** Expression levels of the putative ceRNA genes in 5q- samples compared with the controls

Gene name	Mean 5q-	SD 5q-	SE 5q-	Mean CNTR	SD CNTR	SECNTR	p-value	p <0.05
DCX	11.62274	1.82978	0.33978	10.91179	1.54032	0.37358	**0.18543**	
GRAMD1B	173.15608	48.10625	8.93311	217.83253	40.57191	9.84013	**0.00245**	**DOWN**
HIPK2	270.99334	102.12731	18.96457	213.90735	66.42774	16.11109	**0.04549**	**UP**
SLC1A2	15.62593	2.33554	0.4337	16.00038	2.37252	0.57542	**0.60439**	

A comparison of the normalized expression levels, as reported in GDS3795 dataset, of the putative ceRNA genes between 5q- patients and controls. The genes that showed in a student’s t-test a statistically significant (p<0.05) difference of expression levels are considered as positive results. SE= Standard Error. SD= Standard Deviation. CNTR= Controls.

*GRAMD1B* codes for a protein involved in chemoresistance [66] and the rs735665 SNP upstream of its coding sequence has been associated with chronic lymphocytic leukemia (CLL) in a genome-wide association study [67, 68].

HIPK2 is part of the AML1 complex, and it activates its transcriptional activity. Noteworthy, AML1 is a frequent target of leukemia-associated mutations. It has been reported that *HIPK2* mutations in AML and MDS impair AML1-mediated transcription. It has been therefore suggested that a deregulation of *HIPK2* may play a role in the pathogenesis of leukemia [69].

The results obtained through the approach prospected herein have, however, some limitations. Indeed, the algorithms used to identify the interactions between transcripts and microRNAs are still imperfect. Even if the criterion used was highly stringent (i.e. the contemporary detection by 4 of the most used algorithms) the certainty of the result is far from being achieved. The adoption of different algorithms and/or different parameters could lead to different results.

Similarly, the adoption of a different, and less stringent threshold in the selection of microRNAs could have led to different results.

Finally, the interpretation of the results is rather complex. If microRNAs can act only as inhibitors of transcription and translation, the ceRNAs isolated through this analysis should have been consistently downregulated. Instead, the analysis identified *HIPK2* that is significantly upregulated in the 5q- patients as compared with controls. It is known that microRNAs can also upregulate the transcription [70-72], and maybe that is the case. Alternatively, HIPK2 upregulation could be the result of complex perturbations of the RNA regulatory network. Nevertheless, the analysis that we prospected was able to pinpoint two genes significantly modulated in patients, as compared with controls, and whose relationship with 5q- deletion was never reported before.

The method prospected here represents a novel approach to study the global effects of genomic deletions with the final aim of identifying unexpected contributors to the genomic deletion phenotypes and could deserve experimental validation. The same approach might be used to study duplications or complex rearrangements, leading to a new strategy to question complex syndromes and phenotypes that at the moment are not fully understood.

## MATERIALS AND METHODS

The published GDS3795 affymetrix array dataset [65] was used to select the genes in haploinsufficiency within the common 5q deleted region [73] in bone marrow CD34+ cells of myelodysplastic syndrome patients. We selected patients that showed only 5q- deletion without other reported rearrangements. In detail, we selected 29 patients (MDS patient 4, 5, 6, 7, 8, 10, 11, 12, 14, 15, 21, 23, 24, 26, 31, 35, 36, 45, 58, 62, 70, 90, 95, 109, 121, 132, 135, 154, 168) and compared them with the 17 healthy controls of the dataset (Supplemental data).

The expression levels of the genes within the common deleted region between 5q- patients and controls were compared. The genes that showed in a student’s t-test a statistically significant (p < 0.05) reduction of expression levels in 5q- specimens were considered as haploinsufficient genes (Table 1). As expected, no genes showed an increase in expression levels.

The miRWalk 2.0 [74] platform was used to identify the microRNAs that putatively regulate the genes in haploinsufficency. We considered as positives the microRNAs that recognize the 3’UTR of the genes, with a minimum seed length of 7 and from miRNA seed position 1, with a maximum of p-value of 0.05 in all of the 4 algorithms embedded in the platform used during the analysis: miRWalk [74], miRanda [75], RNA22 [76] and TargetScan [77]. If a gene was recognized multiple times by the same microRNA, it was considered as a single hit in the following analyses. This collection of microRNAs was then organized in a hierarchical order from the most present to the less, and only microRNAs that putatively regulated 5 or more genes in haploinsufficiency were selected for the following analyses. The threshold of 5 was selected to harvest a sufficient number of microRNA to continue the analysis, ideally in the range of the number of microRNAs that can control a single gene, from 4 to 20 [78] (Supplemental data).

Using the miRWalk 2.0 platform with the same parameters described above, the genes putatively regulated by this pool of microRNAs were identified, and those genes that resulted regulated by all the microRNAs were selected. The expression levels of the candidate genes were then analyzed in the same samples from the GDS3795 dataset.
